# Treatment Planning of Bulky Tumors Using Pencil Beam Scanning Proton GRID Therapy

**DOI:** 10.14338/IJPT-22-00028

**Published:** 2022-12-22

**Authors:** Aditya Halthore, Zachary Fellows, Anh Tran, Curtiland Deville, Jean L. Wright, Jeffrey Meyer, Heng Li, Khadija Sheikh

**Affiliations:** 1 Department of Radiation Oncology and Molecular Sciences, Johns Hopkins University School of Medicine, Baltimore, MD, USA; 2 Department of Radiation Oncology, The Johns Hopkins Proton Center, Washington, DC, USA

**Keywords:** proton, spatial fractionation, GRID, bulky tumors

## Abstract

**Purpose:**

To compare spatially fractionated radiation therapy (GRID) treatment planning techniques using proton pencil-beam-scanning (PBS) and photon therapy.

**Materials and Methods:**

PBS and volumetric modulated arc therapy (VMAT) GRID plans were retrospectively generated for 5 patients with bulky tumors. GRID targets were arranged along the long axis of the gross tumor, spaced 2 and 3 cm apart, and treated with a prescription of 18 Gy. PBS plans used 2- to 3-beam multiple-field optimization with robustness evaluation. Dosimetric parameters including peak-to-edge ratio (PEDR), ratio of dose to 90% of the valley to dose to 10% of the peak VPDR(D90/D10), and volume of normal tissue receiving at least 5 Gy (V5) and 10 Gy (V10) were calculated. The peak-to-valley dose ratio (PVDR), VPDR(D90/D10), and organ-at-risk doses were prospectively assessed in 2 patients undergoing PBS-GRID with pretreatment quality assurance computed tomography (QACT) scans.

**Results:**

PBS and VMAT GRID plans were generated for 5 patients with bulky tumors. Gross tumor volume values ranged from 826 to 1468 cm^3^. Peak-to-edge ratio for PBS was higher than for VMAT for both spacing scenarios (2-cm spacing, *P* = .02; 3-cm spacing, *P* = .01). VPDR(D90/D10) for PBS was higher than for VMAT (2-cm spacing, *P* = .004; 3-cm spacing, *P* = .002). Normal tissue V5 was lower for PBS than for VMAT (2-cm spacing, *P* = .03; 3-cm spacing, *P* = .02). Normal tissue mean dose was lower with PBS than with VMAT (2-cm spacing, *P =* .03; 3-cm spacing, *P =* .02). Two patients treated using PBS GRID and assessed with pretreatment QACT scans demonstrated robust PVDR, VPDR(D90/D10), and organs-at-risk doses.

**Conclusions:**

The PEDR was significantly higher for PBS than VMAT plans, indicating lower target edge dose. Normal tissue mean dose was significantly lower with PBS than VMAT. PBS GRID may result in lower normal tissue dose compared with VMAT plans, allowing for further dose escalation in patients with bulky disease.

## Introduction

Spatially fractionated radiation therapy, also known as GRID therapy, delivers high-dose radiation to small volumes in a manner that creates alternating regions of high and low dose within gross disease [1]. The motivation for using alternating regions of high and low dose in place of a regular large, homogeneous field comes from the clinical observation that normal tissues surrounding or adjacent to tumors can tolerate a higher dose if the radiation is concentrated in a small volume [2]. Biologically, spatially fractionated high-dose radiation in tumor may elicit bystander and abscopal effects and have different effects on the tumor microenvironment compared with conventional whole-volume irradiation [3]. The standard delivery method for GRID involves the use of collimating blocks with a distinctive transmission pattern mounted on a linear accelerator. A single 15- to 20-Gy fraction is followed by a traditional course of treatment [4]. More recent GRID delivery methods have used multileaf collimator modulation, volumetric modulated arc therapy (VMAT), or tomotherapy [5–7]. However, delivery of high-dose GRID therapy may not be possible with the above techniques, particularly in situations where critical organs lie adjacent to tumor and/or the tumors are particularly remote from the skin surface. Pencil-beam scanning (PBS) proton therapy has been used to improve dose distributions in proof-of-concept models, dosimetry studies [8, 9], and in the clinical setting with a sample size of 10 patients [10]. These methods used single-field PBS with spot pattern mimicking the brass collimation block, which historically has been the most commonly used modality in the clinical setting [4, 11]. This method results in a significant portion of high dose delivered superficially, thereby limiting its utility for deeper tumors. A solution, which maintains the unique geometry of high-dose “islands” within the tumor, is to dose paint 3-dimensional target structures along the axis of the tumor. This approach has been studied using VMAT and tomotherapy [7]. The objectives of this study were to compare the dosimetry of VMAT with a PBS-based GRID approach using cylindrical target structures in 5 sample patients, and to evaluate the robustness of PBS-based GRID in 2 clinical patients treated on an ongoing clinical trial (NCT05121545) [12].

## Materials and Methods

**Figure 1.**
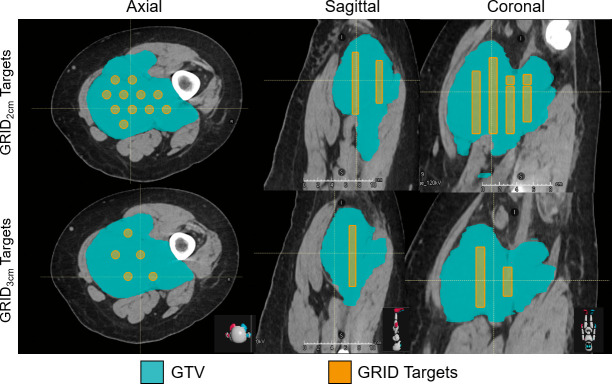
GRID target (orange) orientation with 2-cm (top) and 3-cm (bottom) spacing overlaid on CT in axial, coronal, and sagittal planes. The GTV is shown in cyan. Abbreviations: CT, computed tomography; GRID, spatially fractionated radiation therapy; GTV, gross tumor volume.

In 2 institutional review board (IRB)-approved protocols, CT scans from 5 patients with bulky tumors (≥7 cm in diameter) were obtained, and 2 patients were treated using PBS-based GRID therapy. For each scan, gross tumor volume (GTV) was contoured along with normal organs at risk (OAR) in RayStation 10A (RaySearch, Stockholm, Sweden). For the sample plans, cylindrical targets measuring 1 cm in diameter within the GTV were created using DICOMan (University of Arkansas for Medical Sciences, Little Rock, Arkansas) and arranged longitudinally along the long axis of tumor (**Figure 1**) [13]. Target edges were arranged 8 mm from GTV edge in the left-right and anterior-posterior directions. Similar to previous work that investigated various photon GRID methods, plans were created for targets spaced at 2 cm [14] and 3 cm [7, 8]. **Supplemental Table S1** shows dose objectives for the targets; objectives were included to minimize the OAR dose, which depended on the anatomic site and geometry. For the VMAT plans, 2 full arcs of 6-MV photons were used to deliver treatment to targets within the GTV. PBS plans (both 2- and 3-cm GRID spacing) were optimized using multiple-field optimization. A range shifter was used to treat tumor with depths less than 4 cm. Each plan was evaluated under robust conditions with a range uncertainty (RU) of 3.5% and setup uncertainty (SU) of 2 mm. The worst-case scenarios estimated for RU and SU were determined. In addition, we robustly optimized and evaluated using RU of 3.5% and SU of 5 mm in all directions to evaluate the robustness of 2 mobile targets (liver and right psoas tumors). The maximum edge dose (Edge_max_) and mean edge dose (Edge_mean_) were evaluated. **Supplemental Table S2** summarizes the beam arrangements used for the PBS plans.

We computed multiple dosimetric values to compare the photon and PBS-GRID plans (**Supplemental Table S3**). Briefly, the mean peak dose was defined as the mean dose to the cylindrical GRID target volumes. The mean edge dose was defined as the mean dose to a 2-mm ring exterior to the GTV, and the maximum edge dose as the maximum dose to this ring. The peak-to-edge ratio (PEDR) was defined as the ratio of the mean peak dose to the mean edge dose. The peak-to-valley ratio (PVDR) was defined as the ratio of the mean peak dose to the mean dose to the valley volume. Furthermore, using the GRID/LATTICE therapy guidelines from Zhang et al [15], we determined the valley-to-peak dose ratio VDPR(D90/D10) as the ratio of the D90 dose to the valley to the D10 dose to the GRID target volumes. We also evaluated the dose density, which is the ratio of the volume of the GTV receiving 18 Gy or more to the volume of the GTV. Finally, we evaluated dose to the normal tissues (all tissues 2 cm external to the GTV) by measuring their V5 Gy and V10 Gy values (volume of normal tissue receiving 5 Gy or more and 10 Gy or more, respectively). All proton doses were reported in terms of biologic dose (factor of 1.1). Although, the biologic effects remain to be explored, the dose distribution evaluation parameters were chosen based on the Physics Working Group in GRID/LATTICE therapy [15].

We generated clinical PBS-GRID plans in 2 patients with bulky disease. Both patients consented to an IRB-approved phase 1 clinical trial investigating proton GRID therapy. Cylindrical targets measuring 1 cm in diameter within the GTV were created using an in-house script using RayStation. Similar to the sample comparative patient plans, the treatment plans were optimized by multiple-field-optimization using robust optimization accounting for RU (3.5%) and SU (2 mm). The robustness of the PBS-GRID plans was determined using the quality assurance computed tomography (QACT) scans, which were acquired 1 day prior to treatment. All GRID targets were treated to a prescription of 18 Gy in a single fraction. As a comparison, the PBS-GRID_3cm_ plans were generated using a single field. Patient-specific quality assurance (QA) was performed using the Octavius phantom (PTW, Freiburg, Germany) for the clinical plans. Measurements were acquired in the coronal plane at depths of 2 and 4 cm depending on the range of the beam being evaluated. The criteria for distance-to-agreement (DTA) and dose difference were 2%/2 mm. A 10% threshold of maximum dose was applied, and γ pass rates were) computed. A pass rate was considered to be above 95%. Data are available upon request.

Data were tested for normality using the Shapiro-Wilk normality test using SPSS 26.0 (IBM, Armonk, New York). Paired *t* tests with a Holm-Bonferroni correction for multiple comparisons were used to compare measurements. Results were considered significant when the probability of making a type I error was less than 5% (*P* < .05).

## Results

**Figure 2.**
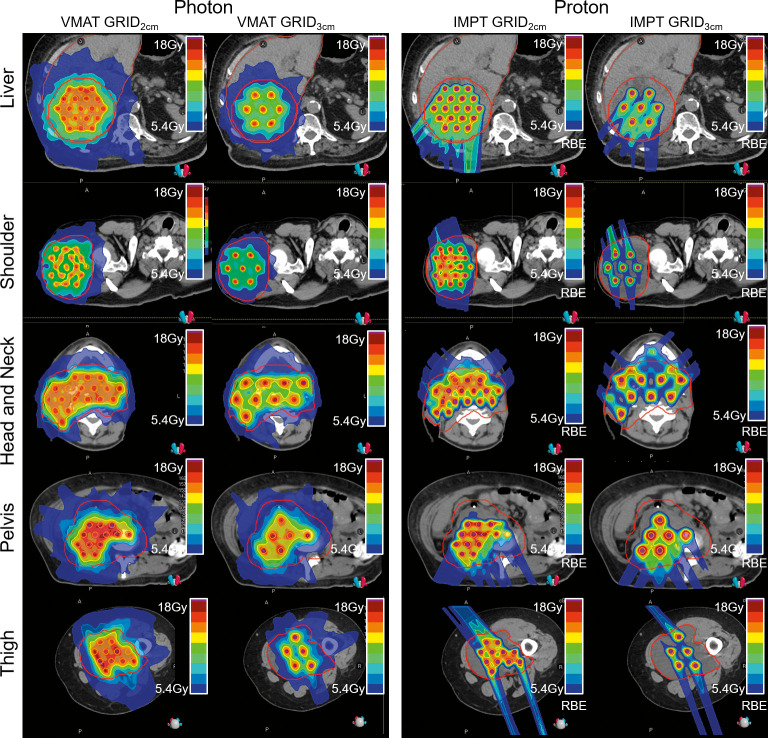
Representative VMAT (left) and IMPT (right) plans are shown with GRID targets at 2- and 3-cm spacing for all 5 planning cases. The GTV is shown in red. Abbreviations: CT, computed tomography; GRID, spatially fractionated radiation therapy; GTV, gross tumor volume; IMPT, intensity modulated proton therapy; VMAT, volumetric modulated arc therapy.

**Figure 2** displays representative dose distributions on axial slices for VMAT and PBS plans at 2-and 3-cm spacing for all 5 cases. It is visually apparent that the proton plans resulted in a lower dose outside of the target. In contrast, the photon VMAT plans show higher low-dose spillage outside of the target (540 cGy isodose line). The 2-cm spaced GRID plans show higher valley dose compared with the 3-cm spaced plans. It should be noted that the sample proton plans were created using 2 beams and visually demonstrate a higher D_max_ at the edge of the GTV.

**Table 1** shows the mean dose distribution parameters for the targets and normal tissues. Briefly, the non-target volume receiving 5 Gy or more (V5) was significantly lower for PBS when compared with VMAT (2-cm spacing, *P =* .03; 3-cm spacing, *P =* .02). The non-target V5 was not significantly different when 2-cm and 3-cm spacing were compared for the same modality. The non-target volume receiving 10 Gy or more (V10) was not significantly different when comparing PBS and VMAT, or when 2-cm and 3-cm spacing were compared within the same modality. Mean dose to normal tissue beyond GTV was significantly lower using PBS when compared with VMAT (2-cm spacing, *P =* .03; 3-cm spacing, *P =* .02). Mean dose beyond GTV did not differ when 2-cm and 3-cm spacing were compared for each modality.

**Table 1.** Comparison of mean target and normal tissue parameters across all 5 patient plans for VMAT or PBS at 2- and 3-cm spacing.

	**VMAT GRID_2 cm_**	**VMAT GRID_3 cm_**	**PBS GRID_2 cm_**	**PBS GRID_3 cm_**
Target parameters				
GRID D_mean_ (Gy)	18.0 ± 0.43	18.4 ± 0.4	19.3 ± 0.6	19.7 ± 0.3
GRID D_max_ (Gy)	19.9 ± 0.2	20.3 ± 0.8	23.2 ± 1.7	22.2 ± 1.4
GRID D_95_ (Gy)	16.9 ± 0.8	17.0 ± 0.8	17.2 ± 1.1	17.9 ± 0.8
GTV D_mean_ (Gy)	12.9 ± 1.8	10.9 ± 1.1	10.0 ± 2.0	7.2 ± 1.4
GTV Edge D_mean_ (Gy)	7.7 ± 1.6	6.6 ± 0.8	2.9 ± 0.8	2.4 ± 0.6
GTV Edge D_max_(Gy)	15.6 ± 2.8	14.0 ± 3.0	15.6 ± 3.1	15.9 ± 1.6
PEDR^a^	2.4 ± 0.6	2.8 ± 0.4	7.2 ± 2.5	8.4 ± 2.0
PVDR^a^	1.5 ± 0.2	1.7 ± 0.2	2.4 ± 0.5	3.1 ± 0.8
Dose density^b^	0.08 ± 0.04	0.03 ± 0.01	0.11 ± 0.03	0.06 ± 0.02
VPDR(D90/D10)^a^	0.41 ± 0.12	0.33 ± 0.08	0.03 ± 0.03	0.01 ± 0.01
GTV D5 (Gy)^c^	18.2 ± 0.4	17.4 ± 0.5	19.4 ± 0.7	15.4 ± 0.5
GTV D10 (Gy)^d^	17.8 ± 0.4	15.9 ± 0.8	18.4 ± 0.4	13.3 ± 1.6
GTV D20 (Gy)	16.8 ± 0.7	14.2 ± 1.2	16.3 ± 1.0	10.3 ± 2.2
GTV D50 (Gy)^c^	13.4 ± 2.6	10.7 ± 1.4	9.4 ± 3.4	5.4 ± 2.4
GTV D90 (Gy)^a^	7.7 ± 2.3	6.4 ± 1.3	0.8 ± 0.8	0.2 ± 0.1
Normal tissue parameters				
D_mean_ (Gy)^a^	1.1 ± 0.5	1.0 ± 0.4	0.2 ± 0.2	0.2 ± 0.1
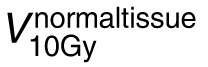 (cm^3^)	21 ± 29	10 ± 12	15 ± 17	8 ± 6
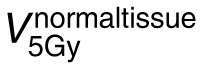 (cm^3^)^a^	1101 ± 551	624 ± 217	411 ± 211	300 ± 138

a statistically significant (*P <* .05) difference between VMAT and PBS for both spacings.

b statistically significant (*P <* .05) difference between PBS 2- and 3-cm spacings.

c statistically significant (*P <* .05) difference between VMAT and PBS for both spacings, between PBS 2- and 3-cm spacings, and between VMAT 2- and 3-cm spacings.

d> statistically significant (*P <* .05) difference between VMAT 2- and 3-cm spacings.

**Abbreviations:** VMAT, volumetric modulated arc therapy; GRID, spatially fractionated radiation therapy; PBS, pencil-beam scanning; D_mean_, mean dose of structure; D_max_, maximum dose to structure; GTV, gross tumor volume; PEDR, peak-to-edge dose ratio; PVDR, peak-to-valley dose ratio; VPDR(D90/D10), ratio of valley D_90_ to peak D_10_; 
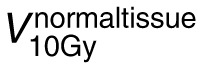
, volume of normal tissue receiving at least 10 Gy; 
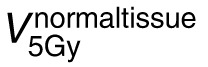
, volume of normal tissue receiving at least 5 Gy.

**Figure 3.**
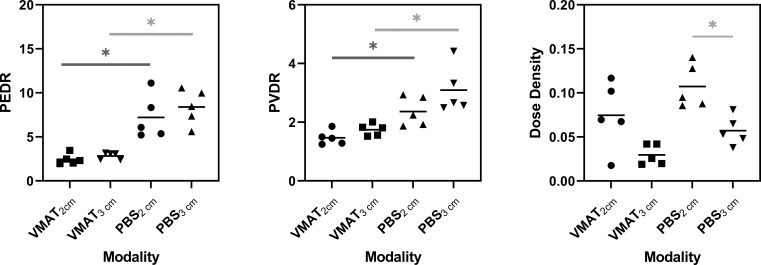
PEDR, PVDR, and dose density for all cases for VMAT and PBS techniques at 2- and 3-cm spacing (n = 5). Asterisked connectors show statistically significant (P < .05) difference between means. Abbreviations: PBS, pencil-beam scanning; PEDR, peak-to-edge dose ratio; PVDR, peak-to-valley dose ratio; VMAT, volumetric modulated arc therapy.

The PEDR, PVDR, and dose density for each patient according to modality and spacing are shown in **Figure 3**. The PEDR for PBS was significantly higher when compared with VMAT (2-cm spacing, *P =* .02; 3-cm spacing, *P =* .01). There were no significant differences in PEDR when 2-cm and 3-cm spacing were compared within the same modality. PVDR was significantly higher for PBS when compared with VMAT (2-cm spacing, *P =* .02; 3-cm spacing, *P =* .03). There were no significant differences in PVDR when 2-cm and 3-cm spacing were compared within the same modality. Mean dose density was significantly higher in the 2-cm spacing PBS plans when compared with that of the 3-cm spacing PBS plans. GRID D_mean_ was higher for PBS plans when compared with VMAT plans in each of the 5 cases. It should be noted that the mean target dose for PBS plans averaged together was not significantly higher than that of the VMAT plans for this sample size. Similarly, GRID D_max_ was appreciably higher for PBS plans in every case, but when averages were compared, the maximum target dose differences for PBS and VMAT plans at 2-cm and 3-cm spacing were not statistically significant.

The worst-case scenarios for RU and 3-mm setup uncertainty were evaluated for the PBS GRID_2cm_ and GRID_3cm_ treatment plans (**Table 2**). The worst-case PVDR was significantly higher in the PBS GRID_3cm_ treatment plans (GRID_2cm_: 2.0 ± 0.5, vs GRID_3cm_: 3.0 ± 0.8; *P* = .01), suggesting that the valley D_mean_ was robust to over- and under-range estimations in addition to 3-mm shifts. No differences were observed in the worse cases scenarios for GTV Edge D_mean_ (*P* = .1) or GTV Edge D_max_ (*P* = .8). There were no significant differences observed in the worst-case normal tissue dose parameters, specifically the normal tissue D_mean_ (*P* = .1), 
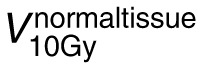
 (*P* = .2), or 
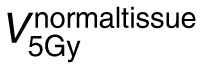
 (*P* = .1), suggesting both spacings allow for robust treatment plans in terms of GTV edge and normal tissue doses. The RU and 5-mm setup uncertainty were evaluated for the proton plans with mobile tumors (**Supplemental Table S4**). The max edge dose, PVDR, and VPDR(D90/D10) are shown for the plans that were (liver–robust optimized [RO] and psoas-RO) and were not (liver and psoas) optimized robustly. In general, the 2-cm spaced RO plans had a lower maximum edge dose compared with the 3-cm spaced RO plans. When evaluating the RU for all 5 patients, there were no significant differences in PEDR in the scenarios of over- and under-ranging (3.5%). This may be due to the 8-mm margin that was created between the GRID targets and the GTV as described in the Materials and Methods section.

**Table 2.** Comparison of worst-case robustness scenarios (RU: 3.5%, SU: 3mm) for mean target and normal tissue parameters across all 5 patient plans planned with PBS at 2- and 3-cm spacing.

	**PBS GRID_2cm_**	**PBS GRID_3cm_**
Target parameters		
GRID D_mean_ (Gy)	18.4 ± 0.5	18.6 ± 0.2
GRID D_max_ (Gy)	24.1 ± 1.3	22.9 ± 2.6
GRID D_95_ (Gy)	15.5 ± 4.3	14.5 ± 1.4
Valley D_mean_ (Gy)^a^	9.4 ± 1.9	6.4 ± 1.6
GTV D_mean_ (Gy) ^a^	9.4 ± 1.9	6.9 ± 1.4
GTV Edge D_mean_ (Gy)	3.0 ± 0.8	2.5 ± 0.7
GTV Edge D_max_(Gy)	16.4 ± 3.5	15.9 ± 1.3
PEDR	6.7 ± 2.3	7.7 ± 1.8
PVDR ^a^	2.0 ± 0.5	3.0 ± 0.8
VPDR(D90/D10)	0.04 ± 0.03	0.01 ± 0.01
GTV D5 (Gy)	19.1 ± 0.7	17.9 ± 0.9
GTV D10 (Gy)^a^	15.1 ± 0.5	15.4 ± 1.8
GTV D20 (Gy)^a^	13.5 ± 0.8	12.0 ± 2.3
GTV D50 (Gy)^a^	7.5 ± 3.4	6.2 ± 2.3
GTV D90 (Gy)	0.6 ± 0.7	0.1 ± 0.1
Normal tissue parameters		
D_mean_ (Gy)	0.2 ± 0.1	0.2 ± 0.1
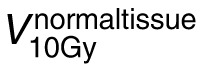 (cm^3^)	19 ± 18	7 ± 6
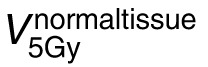 (cm^3^)	428 ± 210	274 ± 185

a statistically significant (*P <* .05) based on paired *t* test.

**Abbreviations:** PBS, pencil-beam scanning; GRID, spatially fractionated radiation therapy; D_mean_, mean dose of structure; D_max_, maximum dose to structure; GTV, gross tumor volume; PEDR, peak-to-edge dose ratio; PVDR, peak-to-valley dose ratio; VPDR(D90/D10), ratio of valley D_90_ to peak D_10_; 
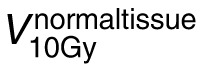
, volume of normal tissue receiving at least 10 Gy; 
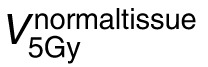
, volume of normal tissue receiving at least 5 Gy.

**Figure 4.**
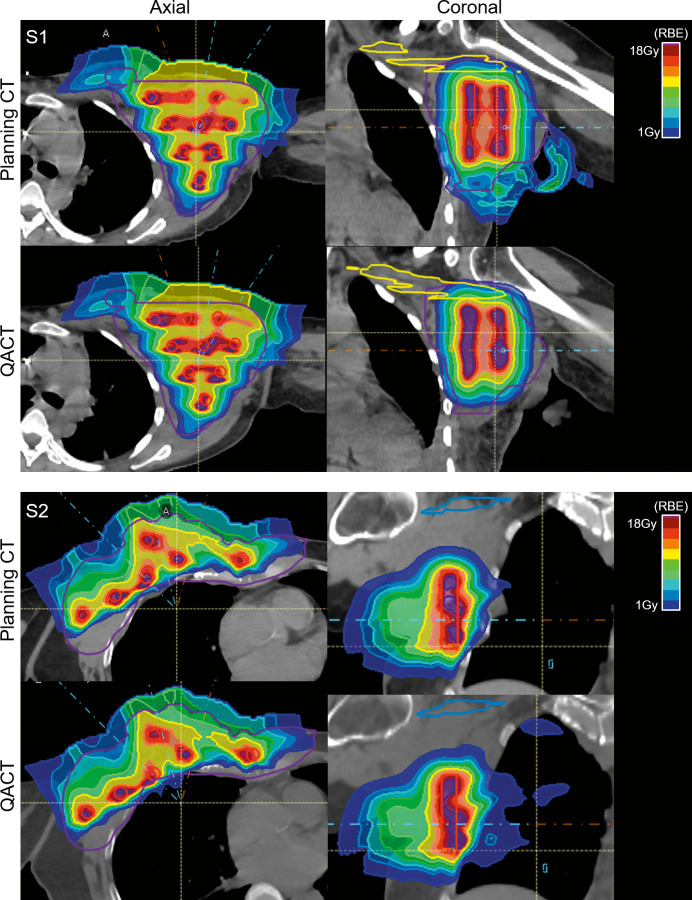
PBS-GRID plans for 2 patients (S1 and S2) enrolled onto a prospective phase 1 clinical trial evaluating the feasibility of proton GRID for the treatment of bulky tumors. PBS GRID with 3-cm center-to-center spacing was used. Plan computed on the planning CT (top) and on the QACT 1 day prior to treatment (bottom) shown in the axial and coronal planes. S1: purple contour indicates GTV, yellow contour indicates brachial plexus (the dose limiting organ), and red indicates the GRID targets. S2: purple contour indicates the GTV, blue contour indicates the brachial plexus, and red contours indicate the GRID targets. Abbreviations: CT, computed tomography; GRID, spatially fractionated radiation therapy; GTV, gross tumor volume; PBS, pencil-beam scanning; QACT, quality assurance CT.

Based on the increased PEDR and PVDR observed in the PBS_3cm_ treatment plans, we generated 2 clinical plans using 3-cm center-to-center spacing. **Figure 4** shows PBS-GRID plans for 2 patients (S1 and S2) enrolled onto a prospective phase 1 clinical trial. The plan computed on the planning CT is shown on the top and the plan computed on the QACT is shown on the bottom. For both patients, the QACT was acquired 5 days after the planning CT; this corresponded to the day prior to treatment. We evaluated the PEDR, PVDR, VPDR(D90/D10), and the dose to the dose-limiting structures. In both cases (S1/S2), the PEDR was 4.11/3.53 at the time of planning and 3.71/3.41 at the time of QACT. In both cases, the PVDR was 2.04/2.02 at the time of planning and 2.11/1.84 at the time of QACT. The VPDR(D90/D10) was 0.05/0.06 at the time of planning and 0.02/0.1 at the time of QACT. For both patients (S1/S2), the max dose to the skin was 13.2 Gy(RBE)/17.5 Gy(RBE) at the time of planning and 14.9 Gy(RBE)/16.8 Gy(RBE) at the time of QACT. For S1, the max dose to the brachial plexus was 7.8 Gy(RBE) at the time of planning and 11.8 Gy(RBE) at the time of QACT. The increase in brachial plexus dose may be a result of the tumor growth observed between CT simulation and treatment, which consequently modified the patient arm position (shown in **Figure 4**). However, the doses to the dose-limiting structures were consistent with the single fraction dose limit recommendations outlined in the report by Benedict et al [16] and deemed acceptable for treatment. Finally, we must note that S2 had a reduction in the anterior-posterior thickness across the right breast of 3.4 cm (7.2 to 3.8 cm) and lobular mass reduction of 2.4 cm (11.3 to 8.9 cm) 3 weeks following proton GRID. If treated as a sphere, (initial radius: 7.2 cm; initial volume: 162 cm^3^; post-GRID radius: 3.8 cm; post-GRID volume: 45 cm^3^) this translates to a decrease of 70% in volume.

We compared the PEDR, PVDR, and VPDR(D90/D10) for single-field versus multifield PBS_3cm_ treatment plans (**Supplemental Table S5**). In brief, the PEDR and PVDR were significantly higher in the multifield PBS_3cm_ plans (*P* < .05); whereas, the volume of normal tissue receiving 10 Gy was significantly higher in the single-field plans (*P* = .01). When comparing the mean edge dose of the single-field plans that used range shifters (5.4 ± 0.8 Gy) versus no range shifters (2.9 ± 0.7 Gy), it was apparent that the mean edge dose was significantly higher in patients requiring range shifters (*P* = .01). The mean valley dose was significantly higher in patients requiring range shifters (*P* = .02) in the single-field optimized plans (range shifters: 10.5 ± 0.5 Gy vs no range shifters: 7.6 ± 1.6 Gy). These differences were not observed in the multifield plans.

Patient QA was completed for patients S1 and S2 prior to treatment. The calculated measured dose distributions along with the γ for all beams (S2) are shown in the **Supplemental Figure**. Briefly, the γ map pass rates (2%/2 mm%) were 100%, 95%, and 100% for beams 1, 2, and 3, respectively. A depth of 2 cm was used for all beams. For S1, the γ map pass rates (2%/2 mm%) were 95%, 96%, and 100% for beams 1, 2, and 3, respectively. A measurement depth of 2 cm was used for beams 1 and 3, and a depth of 4 cm for beam 2.

## Discussion

In this study, to better evaluate GRID treatment planning techniques, we investigated the dose distributions of PBS and VMAT GRID treatment plans of bulky disease. We made the following observations: (1) the valley and edge doses were significantly lower using PBS compared with VMAT, (2) the normal tissue dose was significantly lower using PBS compared with VMAT, and, (3) the PBS plans were robust to range and setup uncertainties.

The concept of peak-to-valley ratios and other metrics that describe dose heterogeneity and how they relate to biologic effects remain to be explored. Guidelines set by the Physics Working Group for GRID/LATTICE therapy suggest the evaluation of valley-to-peak ratios along with peak and valley doses [15]. Along these lines, we evaluated the peak-to-valley ratios and demonstrated that the PBS plans, overall, resulted in higher values than the VMAT plans. Conversely, the VPDR(D90/D10) values for the PBS plans were lower than the VMAT plans, indicating that the PBS plans allow for higher GRID target doses compared with the surrounding tissue within the tumor. The biologic consequences of these metrics have not been fully characterized; however, recently published work in a mouse model has illustrated an antitumor immune effect using GRID-treated mice [17]. Specifically, 6 days post irradiation, a significant increase of antigen-presenting cells (eg, dendritic cells, macrophages, and B-cells) were observed in mice treated with GRID therapy versus whole beam irradiation. A recent study also demonstrated an increased therapeutic ratio in glioma-bearing rats irradiated using crossfire proton minibeams with lower valley doses versus a single uniform beam [18]. Although the exact clinical significance of high PVDR values remains to be determined, a higher PVDR may yield a higher therapeutic ratio with better shrinkage and cell kill in bulky tumors while preserving the tolerance of adjacent normal tissue outside the GTV for any subsequent radiation treatment.

It should also be noted that the average PEDR for PBS was lower than for VMAT for every case included in our study, suggesting that PBS planning may reduce regions of low-dose bath outside the GTV when compared with VMAT. This is consistent with other photon GRID studies that have shown dose spillage outside the target [19, 20]. Previous work using proton therapy that has mimicked a photon grid block limits its use to superficial lesions [9, 10]. This is particularly important in the setting of deep-seated tumors, tumors with complex shape, or tumors abutting critical structures. Avoiding dose spillage beyond the gross tumor is also important since these patients with bulky disease may proceed to receive a fractionated course of radiation therapy, during which more dose will be delivered to OARs. There was no difference in PEDR, PVDR, or V5/V10 outside the GTV between 2- and 3-cm spacing within either modality (PBS or VMAT), suggesting that modality rather than spacing has a larger influence on overall GRID quality.

When the peak-to-valley and peak-to-dose ratios were compared between single-field and multifield PBS_3cm_ treatment plans, the multifield plans resulted in higher ratios. This suggests that multifield plans allow for lower valley and edge doses. We also compared the differences between those patients requiring range shifters (ie, shallow tumors) versus those without range shifters (ie, deeper tumors). Single-field range shifter plans resulted in higher edge and valley doses compared with plans without range shifters. Taken together, multifield PBS-GRID plans may offer lower valley and edge doses for both shallow and deep lesions.

Finally, we must acknowledge the range and setup uncertainties observed in PBS treatments. This has not been extensively studied in previously published PBS-GRID studies [9, 10]. While we know that protons are sensitive to intrafractional anatomy motion and setup variations, there is uncertainty in our understanding of where protons stop in patient tissues [21]. For this reason, we evaluated the SU and RU in all patients, and for 2 mobile tumors, we evaluated SU at 5 mm. To further account for setup, we placed cylindrical targets no less than 8 mm from GTV edge. Dose volume histogram differences as well as Edge_max_ and Edge_mean_ in our cohort were not meaningfully altered after accounting for over- or under-ranging of 3.5%. We also evaluated changes in dose distribution after robust optimization for RU and SU in the 2 cases with potentially mobile tumors. Optimizing for RU and SU to account for tumor motion blurred the high-dose regions within the tumor and thereby reduced PVDR in the 2 patients. Taken together, when planning PBS GRID, care must be taken to evaluate RU and SU, especially if cylindrical targets are placed close to the edge of the GTV and in mobile targets.

Although this study compared VMAT- and PBS-GRID planning in a small number of patients, future work is needed to ascertain the clinical and biologic significance of these methods. Our manuscript reflects the technical planning approach that serves as the basis for our currently accruing IRB-approved prospective phase 1 trial. Thus far, we have enrolled 2 patients with bulky breast cancer onto the trial and have successfully delivered the proton GRID treatment in each case. To mitigate setup and range uncertainties, we acquire a QACT prior to treatment for all GRID patients. At the time of treatment, we acquire a cone-beam CT (CBCT) to evaluate the surface of the patient within the beam's path. A repeat CBCT is acquired if shifts are greater than 2 mm/1°. In each case, we have successfully completed the process of CT simulation, QACT just prior to treatment, verification of robustness on QACT, and successful delivery of treatment without incident. We have also completed patient QA successfully for these cases. However, it should also be noted that due to the depth modulation of the PBS-GRID plans, an appropriate depth must be chosen when performing patient QA. A high-dose region will result in poor pass rates. We used 2 depths to ensure the measurement was acquired at a location with a low-dose gradient. We must also note the time from simulation to treatment may be on the order of a week. In the case of urgent palliative cases, PBS-GRID may not be feasible. We have seen a reduction in the tumor size at 3 weeks in a single patient where follow-up was available. We must acknowledge that the first 2 patients enrolled on our trial had superficial lesions; further analysis will evaluate this technique in patients with deep-seated tumors. Recent work has provided recommendations for the use of various GRID techniques [8]. This group has cited that proton therapy has potential clinical application for superficial lesions, pediatric patients, and patients requiring re-irradiation where distal dose must be minimized. Although we do not know at this time if our approach is the optimal way of delivering GRID, we are guided by the physical characteristics of traditional GRID blocks used for photon-based GRID therapy, and we see this as an appropriate starting point [15].

Taken together, this study demonstrated the feasibility of a novel PBS-GRID planning technique. We demonstrated that PBS plans result in higher peak doses while allowing for lower valley and edge doses under robustness. In 2 clinical patients, we demonstrated robust target and critical organ doses using QACTs. Although the biologic significance of the dose heterogeneity parameters is unknown, they will form the basis for future clinical studies. The technical planning approach demonstrated in this manuscript serves as the basis for an ongoing prospective phase 1 trial of proton GRID therapy for bulky malignancies.
