# Supplementary material for: Treatment Planning of Bulky Tumors Using Pencil Beam Scanning Proton GRID Therapy
Source: Int J Part Ther. 2022 Dec 22;9(3):40–9. doi: 10.14338/IJPT-22-00028 (PMC9875826; doi:10.14338/IJPT-22-00028)
Supplement: Supplementary file 1 [file ijpt-09-03-07_s01.docx]

**Supplementary Table 1.** Dose objectives for VMAT and PBS plans.

|  | **Structure** | **Objective** | **Weight** |
| --- | --- | --- | --- |
| ***VMAT*** |  |  |  |
|  | GRID targets | Min dose 1800 Gy | 200 |
|  | GRID targets | Min dose 1800 cGy to 99% volume | 150 |
|  | GRID targets | Max dose 2000 cGy | 25 |
|  | Valley | Max dose 1100 cGy | 50 |
|  | Valley | Dose fall-off: 1100 cGy to 500 cGy at 1 cm distance | 10 |
|  | Normal tissue | Max dose 400 cGy | 10 |
|  |  |  |  |
| ***PBS*** |  |  |  |
|  | GRID targets | Min dose 1800 cGy | 200 |
|  | GRID targets | Min dose 1800 cGy to 95% volume | 80 |
|  | GRID targets | Max dose 2000 cGy | 50 |
|  | Valley | Max 1300 cGy to 5% volume | 30 |
|  | Valley | Max 750 cGy to 20% volume | 30 |
|  | Valley | Dose fall-off: 1200 cGy to 500 cGy at 1 cm distance | 1 |
|  | Valley* | Max dose 1550 cGy | 1000 |

*used for the 2-cm PBS plan (max dose of 1400 with a weight of 10 was used for the 3-cm PBS plans)

**Supplementary Table 2.** Patient characteristics: histology, location, gross tumor volume (GTV) and PBS beam orientation for test plan cases.

| **Tumor Site** | **Histology** | **GTV (cc)** | **PBS Beam Orientation** |
| --- | --- | --- | --- |
| *Sample Patients* | | | |
| Right Shoulder | Undifferentiated pleomorphic sarcoma | 1262 | RAO/LPO |
| Liver | Hepatocellular carcinoma | 998 | PA/RPO |
| Right Thigh | Myxoid liposarcoma | 826 | RAO/LPO |
| Right Psoas | Squamous cell carcinoma of the cervix | 1468 | RPO/LPO |
| Nasopharynx | Pleomorphic sarcoma | 1082 | RAO/LAO |

**Supplementary Table 3.** Dosimetry parameters analyzed and their definitions.

| **Dosimetry Parameter** | **Definition** |
| --- | --- |
| Mean valley dose | Mean dose to GTV minus GRID targets |
| Mean peak dose | Mean dose to GRID targets |
| Mean edge dose | Mean dose to 2 mm ring around GTV |
| Maximum edge dose | Maximum dose to 2 mm ring around GTV |
| Peak-to-edge ratio (PEDR) | $\frac{mean peak dose}{mean edge dose}$ |
| Peak-to-valley ratio (PVDR) | $\frac{mean peak dose}{mean valley dose}$ |
| Dose density | $\frac{volume of 18 Gy isodose line within GTV (cc)}{GTV (cc)}$ |

**Supplementary Table 4.** Nominal plan dosimetric parameters for two PBS plans with mobile tumors (liver and psoas) before and after robust optimization for RU of 3.5% and SU of 5 mm in all directions at 2-cm and 3-cm spacing.

|  | **Liver GRID_2cm_** | **Liver-RO GRID_2cm_** | **Liver GRID_3cm_** | **Liver-RO GRID_3cm_** |
| --- | --- | --- | --- | --- |
| ***Target Parameters*** |  |  |  |  |
| GTV Edge D_max_ (Gy) | 15.7 | 15.6 | 16.3 | 16.4 |
| PVDR | 1.9 | 1.7 | 2.6 | 2.2 |
| VPDR (D90/D10) | 0.05 | 0.09 | 0.007 | 0.01 |
|  |  |  |  |  |
|  | **Psoas GRID_2cm_** | **Psoas-RO GRID_2cm_** | **Psoas GRID_3cm_** | **Psoas-RO GRID_3cm_** |
| ***Target Parameters*** |  |  |  |  |
| GTV Edge D_max_ (cGy) | 15.0 | 15.6 | 14.3 | 14.0 |
| PVDR | 2.8 | 2.7 | 2.7 | 2.6 |
| VPDR (D90/D10) | 0.001 | 0.002 | 0.004 | 0.004 |

D_max_: maximum dose to structure; PVDR: peak-to-valley dose ratio; VPDR (D90/D10): ratio of valley D_90_ to peak D_10_. “Liver-RO”: after robust optimization; “Psoas-RO”: after robust optimization.

**Supplementary Table 5.** Comparison of mean target and normal tissue parameters across all seven patient plans (test plans, n=5; treated plans, n=2) planned with multi-field and single field PBS GRID_3cm_.

|  | **Single Field** | **Multi Field** |
| --- | --- | --- |
| ***Target Parameters*** |  |  |
| GRID D_mean_ (Gy) | 19.3 ± 2.4 | 19.6 ± 0.4 |
| GRID D_max_ (Gy) | 24.5 ± 4.8 | 22.3 ± 1.4 |
| GRID D_95_ (Gy) | 16.4 ± 2.4 | 17.9 ± 0.8 |
| Valley Dmean (Gy) | 8.9 ± 2.2 | 6.6 ± 1.4 |
| GTV D_mean_ (Gy) | 9.5 ± 1.9 | 7.2 ± 1.4 |
| GTV Edge D_mean_ (Gy)* | **4.0 ± 1.6** | **2.5 ± 0.6** |
| GTV Edge D_max_(Gy) | 18.1 ± 6.5 | 16.0 ± 1.5 |
| PEDR* | **5.5 ± 2.1** | **8.4 ± 2.0** |
| PVDR* | **2.3 ± 0.8** | **3.1 ± 0.8** |
| VPDR(D90/D10) | 0.05 ± 0.08 | 0.01 ± 0.01 |
| GTV D5 (Gy) | 18.7 ± 2.2 | 18.5 ± 0.5 |
| GTV D10 (Gy) | 17.3 ± 1.5 | 15.9 ± 1.6 |
| GTV D20 (Gy) | 15.2 ± 1.3 | 12.4 ± 2.1 |
| GTV D50 (Gy) | 10.0 ± 3.8 | 6.4 ± 2.3 |
| GTV D90 (Gy) | 0.9 ± 1.4 | 0.2 ± 0.2 |
|  |  |  |
| ***Normal Tissue Parameters*** |  |  |
| D_mean_ (Gy) | 2.2 ± 0.7 | 2.4 ± 1.2 |
| $V_{10Gy}^{normal tissue}$ (cm^3^)* | **153 ± 63** | **37 ± 14** |
| $V_{5Gy}^{normal tissue}$ (cm^3^) | 280 ± 109 | 246 ± 77 |

*significant difference observed using paired t-test (p<.05)

D_mean_: mean dose of structure; D_max_: maximum dose to structure; PEDR: peak-to-edge dose ratio; PVDR: peak-to-valley dose ratio; VPDR (D90/D10): ratio of valley D_90_ to peak D_10_; $V_{10Gy}^{normal tissue}$: volume of normal tissue receiving at least 10 Gy; $V_{5Gy}^{normal tissue}$: volume of normal tissue receiving at least 5 Gy.
