# Supplementary figures and images for: Treatment Planning of Bulky Tumors Using Pencil Beam Scanning Proton GRID Therapy
Source: Int J Part Ther. 2022 Dec 22;9(3):40–9. doi: 10.14338/IJPT-22-00028 (PMC9875826; doi:10.14338/IJPT-22-00028)

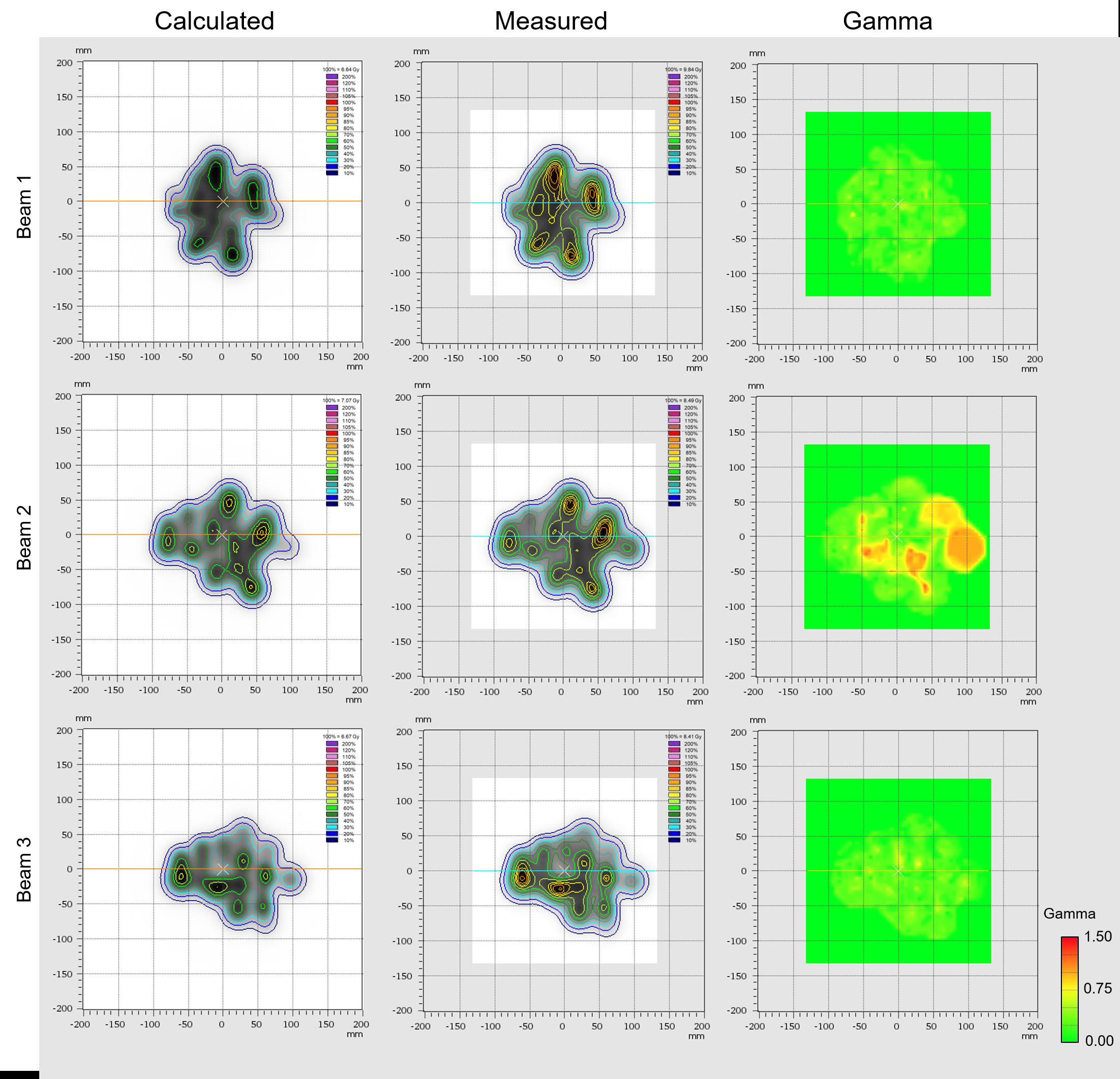

Supplement: Supplementary file 2 [file ijpt-09-03-07_s02.tif]
